# Deletion of exons 17 and 18 in prestin’s STAS domain results in loss of function

**DOI:** 10.1038/s41598-019-43343-y

**Published:** 2019-05-03

**Authors:** Satoe Takahashi, Tetsuji Yamashita, Kazuaki Homma, Yingjie Zhou, Jian Zuo, Jing Zheng, Mary Ann Cheatham

**Affiliations:** 1 Department of Otolaryngology - Head and Neck Surgery, Feinberg School of Medicine, Northwestern University, Chicago, IL USA; 2 St. Jude Children’s Research Hospital, Department of Developmental Neurobiology, Memphis, TN USA; 3 Knowles Hearing Center, Northwestern University, Evanston, IL USA; 4 Roxelyn and Richard Pepper Department of Communication Sciences and Disorders, Northwestern University, Evanston, IL USA; 5 Department of Biomedical Sciences, Creighton University School of Medicine, Omaha, NE USA

**Keywords:** Cochlea, Medical research

## Abstract

Cochlear outer hair cells (OHC) express the motor protein, prestin, which is required for sensitivity and frequency selectivity. Because our previous work showed that a calmodulin binding site (CBS) was located in prestin’s C-terminal, specifically within the intrinsically disordered region, we sought to delete the IDR to study the functional significance of calcium-dependent, calmodulin binding on OHC function. Although the construct lacking the IDR (∆IDR prestin) demonstrated wildtype-like nonlinear capacitance (NLC) in HEK293T cells, the phenotype in ∆IDR prestin knockins (KI) was similar to that in prestin knockouts: thresholds were elevated, NLC was absent and OHCs were missing from basal regions of the cochlea. Although ∆IDR prestin mRNA was measured, no prestin protein was detected. At the mRNA level, both of prestin’s exons 17 and 18 were entirely removed, rather than the smaller region encoding the IDR. Our hybrid exon that contained the targeted deletion (17–18 ∆IDR) failed to splice *in vitro* and prestin protein lacking exons 17 and 18 aggregated and failed to target the cell membrane. Hence, the absence of prestin protein in ∆IDR KI OHCs may be due to the unexpected splicing of the hybrid 17–18 ∆IDR exon followed by rapid degradation of nonfunctional prestin protein.

## Introduction

Although sensory receptor cells are fully differentiated in mammals, it is the outer hair cells (OHC) that are required for cochlear sensitivity and frequency selectivity. Prestin, the OHC motor protein, provides the molecular basis for their ability to function as high-speed voltage sensors. When not expressed, or when motor action is lost, mice suffer an ~50 dB threshold shift^1,2^ and the cochlea no longer functions as a frequency analyzer^3^. Since its discovery^4^, several mouse models have been developed to learn more about prestin’s function. Although prestin is a member of the solute carrier (SLC26) family of anion transporters, prestin’s transport function is minimal. Nevertheless, common structural features are shared by SLC26 family members. For example, prestin has a Sulphate Transporter and an Anti-Sigma (STAS) factor antagonist domain in its C-terminal, which is separated into two parts by an intrinsically disordered region (IDR, amino acids 556–637). Although the function of the STAS domain within the SLC26 family is not fully understood, some data suggest that it may facilitate interactions with other proteins^5,6^. For example, STAS domains of SLC26A3 and SLC26A6 were shown to interact with the R domain of the cystic fibrosis transmembrane conductance regulator (CFTR)^7,8^. Although a functional interaction was also demonstrated between SLC26A5 (prestin) and CFTR using whole proteins^9^, it is not known if the R and STAS domains are mediating this interaction.

In order to learn more about prestin’s regulation, the intracellular C-terminal domain (CTD) was studied by several investigators^10–12^. The CTD, coded by exons 15–20 and extending from amino acids (aa) 505 to 744, includes the STAS domain known to harbor an anion binding site^13^, as well as a motif that interacts with microtubule associated protein 1S (MAP1S)^14^. More recently, prestin’s IDR was shown to contain a calmodulin binding site (CBS)^15^. Because binding of calmodulin to prestin is calcium dependent and the binding serves to shift the operating point of the motor protein to more hyperpolarized potentials, this region has the potential to regulate OHC function. Keller and colleagues also demonstrated that the hyperpolarization of prestin’s voltage dependence was prevented by the calmodulin inhibitor, trifluoperazine (TFP). This previous study also showed that the deletion of the IDR (∆571–635) containing the CBS abrogates the calcium-dependent hyperpolarization. The same effect was obtained using cluster b^11^ where the polarity of six charged amino acids in the region 571–580 were flipped, i.e., basic residues were mutated to aspartates (R571D, R572D, R573D, R576D, K577D, K580D). To maintain consistency, we will refer to cluster b as flipCBS prestin as in our previous publication^15^. Because the efferent neurotransmitter, acetylcholine (ACh), induces an inward calcium current, the CTD of prestin could be influenced by the medial olivocochlear (MOC) reflex^16^. In addition, OHCs have exceptionally abundant calcium binding proteins^17^ and removing the OHC specific calcium binding protein, oncomodulin, leads to dysfunction^18^. Taken together, these results suggest that calcium/calmodulin could directly regulate prestin’s function, although this hypothesis requires verification *in vivo*.

Although several prestin mouse models have been generated^4^, a transgenic mouse designed to study how prestin is regulated has not yet been made. In order to study the functional significance of prestin’s CBS, we endeavored to create an additional prestin knockin (KI) mouse model, adding to the two already in hand: the 499 prestin KI^2^ and the C1 prestin KI mouse^19^. Because the CBS is conserved among mammalian SLC26 family members, it is our hypothesis that calmodulin binding in all SLC26 family members influences function be it transport or, in the case of prestin (SLC26A5), somatic electromotility^20^. If true, this would suggest that the CBS, or more generally the IDR, might be affected in the several human diseases associated with SLC26 family members^15^. Therefore, information obtained from a ∆IDR mouse model could have implications beyond cochlear physiology.

## Results

### *In vitro* characterization of ∆IDR and flipCBS

**Figure 1:**
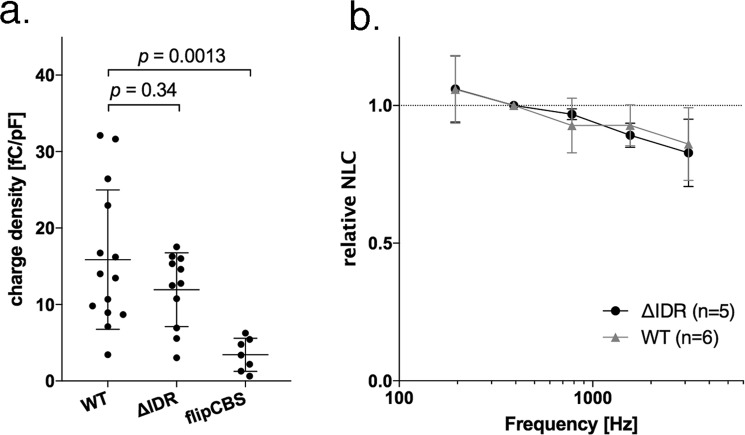
∆IDR prestin retains WT-like nonlinear capacitance. Panel a indicates that HEK293T cells transfected with WT and ∆IDR prestin both exhibit a greater charge density than when transfected with flipCBS. Although the charge density was somewhat larger for WT relative to ∆IDR prestin, the two groups were not statistically different. In contrast, the charge density of flipCBS was statistically lower than for both of the other two groups of transfected cells. Panel b also shows that ∆IDR prestin retains WT-like kinetics. NLC is plotted as a function of the f1 frequency and the data are normalized for f1 = 391 Hz. Error bars represent standard deviations.

Prestin’s C-terminus is known to influence prestin’s structure and to play important roles in membrane targeting^10^. Therefore, to avoid potential membrane targeting problems, we tested two constructs using *in vitro* patch clamp experiments to document nonlinear capacitance (NLC) in cell lines prior to developing a prestin KI mouse model to study the functional significance of prestin’s CBS. For the first design, a region spanning the distal part of exon 17 and the proximal part of exon 18 was targeted for deletion (∆IDR), i.e., amino acids 571–635^15,21^. The second possibility was to use cluster b (flipCBS) in which charged amino acids between 571 and 580 were reversed en bloc. Bai and colleagues^11^ reported that this construct retained NLC but the voltage dependence shifted in the depolarizing direction by ~30 mV. In order to evaluate these two possibilities for manipulating prestin’s calmodulin binding site, we transfected HEK293T cells with ∆IDR and with flipCBS prestin constructs. The charge density for ∆IDR was similar to WT, but that for flipCBS was significantly reduced (Fig. 1a). We also measured and compared various properties of ∆IDR prestin with WT. In addition to retaining its NLC, ∆IDR prestin exhibited WT-like kinetics (Fig. 1b). In summary, ∆IDR prestin proteins are expressed in the plasma membranes of host cells and they have WT-like NLC properties. Because the charge density results imply that there might be a membrane targeting issue or some other factor that decreases the ability of flipCBS prestin protein to insert into the plasma membrane^22^, the decision was made to proceed with the deletion mutant, ∆IDR prestin.

### Creation of the ∆IDR prestin knockin mouse

**Figure 2:**
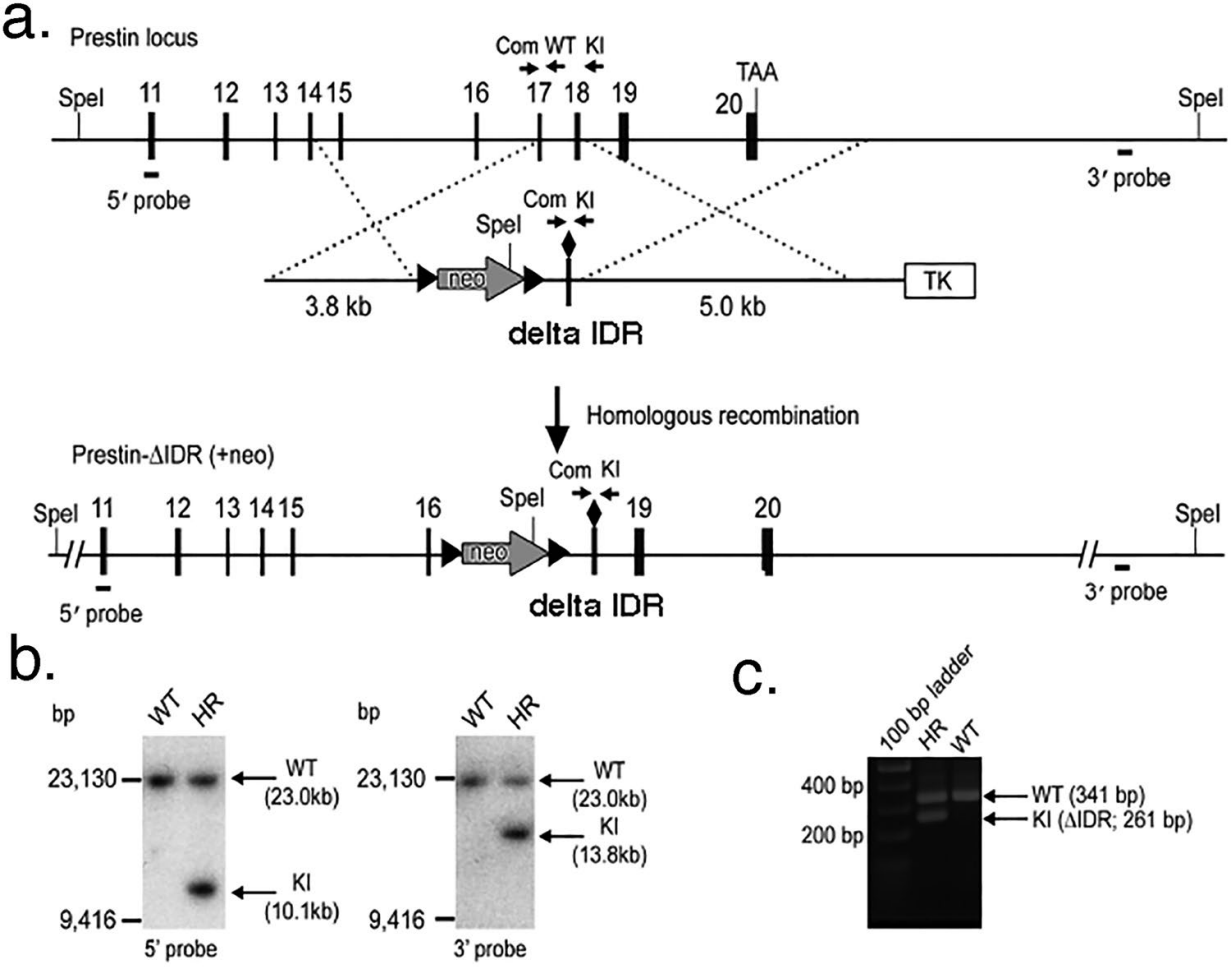
Targeting strategy and analysis of recombinant ES cells. The targeted prestin ∆IDR knockin allele is shown in panel a. Solid vertical rectangles represent exons 11 through 20 of the prestin gene separated by their respective introns. A cassette with the neo-selectable marker flanked by loxP sites (arrowheads) was inserted in the intron between exon 16 and 17 of the *prestin* gene. In addition, the *prestin* gene from exon 17 to 18 was replaced by a hybrid exon lacking a DNA coding region for a portion of the IDR. Genomic Southern blot analysis of prestin ∆IDR homologous recombinant ES cells is provided in panel b. Genomic DNA from wildtype (WT) and homologous recombinant (HR) ES cells was digested separately with SpeI and two specific probes, as indicated in panel a with arrows. PCR-based genomic DNA analysis of wildtype and homologous recombinant ES cells using 3 primers is provided in panel c.

The retention of WT-like kinetics motivated us to develop the ∆IDR prestin KI mouse. Figure 2a shows the prestin locus for exons 11 through 20. The targeting vector was designed to produce hybrid exons 17 and 18 with amino acids 571–635 deleted. The 3.8 and 5.0 kb fragments formed the short and long homologous arms, respectively, of the construct designed for targeting the desired genomic site of prestin exons 17–18 of prestin. The thymidine kinase (*TK*) gene was included for negative selection (ganciclovir, 2 µM) and a floxed neomycin (neo, G418) resistance cassette was inserted before the 17–18 hybrid exon to facilitate ES cell screening using standard protocols described in our previous publications^1,2,19,23,24^. The diamond symbol represents the deletion, 17–18 ∆IDR. Appropriate homologous recombination was confirmed by Southern blot analysis using the restriction enzyme SpeI to digest the genomic DNA into fragments that were then separated via electrophoresis based on their size (Fig. 2b). PCR-based genomic DNA analysis of WT and homologous recombinant cells is provided in Fig. 2c. As expected, both Southern blot and DNA analyses showed the knockin sample, ∆IDR prestin, as a smaller band. The resulting ∆IDR prestin (neo+) mice were then crossed with Ella-Cre mice to remove the loxP-flanked neo cassette.

### Loss of sensitivity in the prestin ∆IDR KI mouse is similar to that in mice lacking prestin

**Figure 3:**
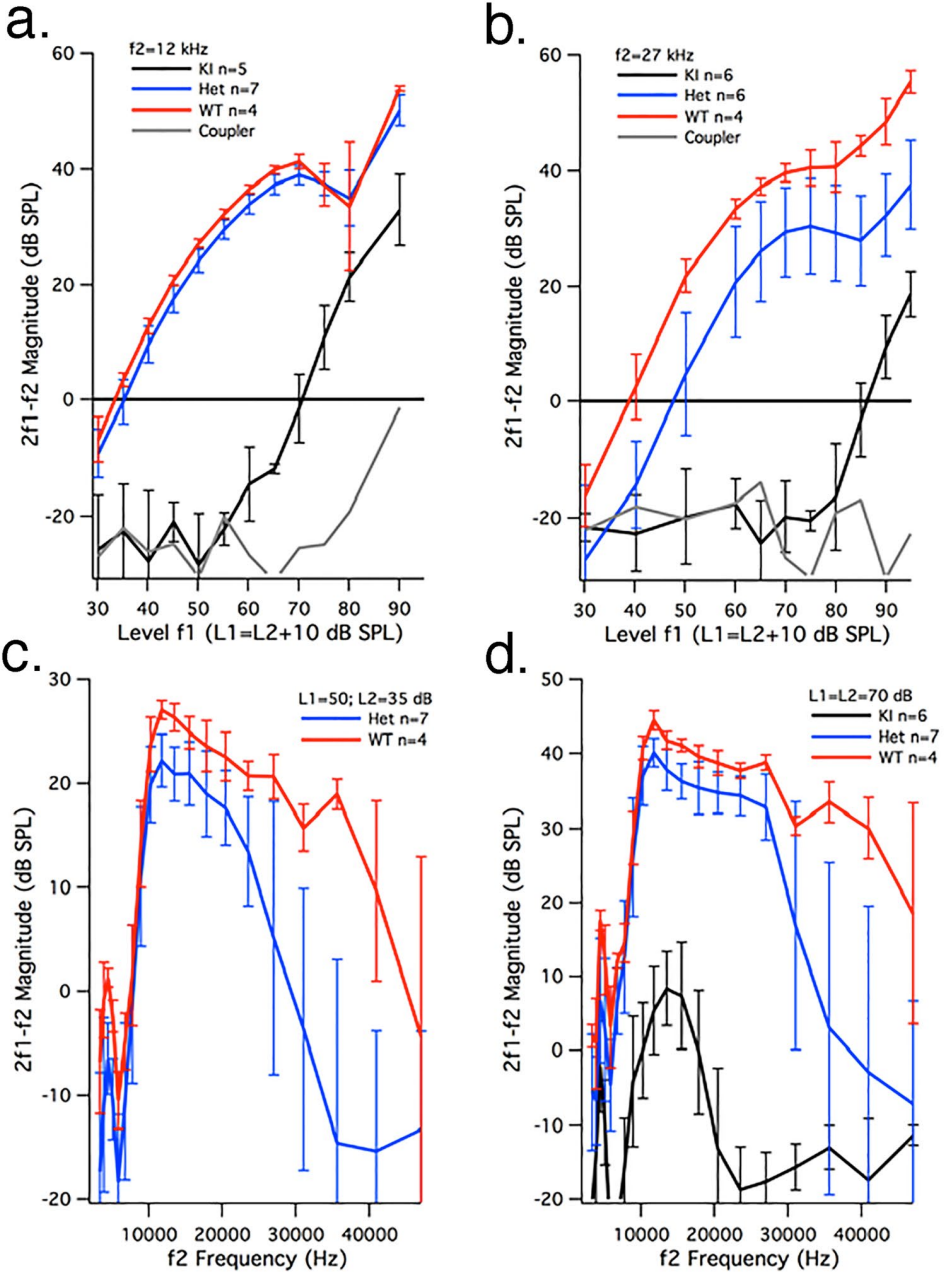
Reduced DPOAEs in the ∆IDR KI mouse. Panel a (**b**) provides average growth functions for f2 = 12 kHz (27 kHz). Although ∆IDR KI mice have smaller DPOAEs than controls, their responses are still larger than the distortion in the sound (coupler), which is plotted in gray. The DPOAE thresholds at 2f1-f2 for f2 = 12 kHz are as follows: WT = 33.2 ± 1.3 dB, n = 4; heterozygotes = 35.2 ± 2.1 dB, n = 7; ∆IDR KIs = 72.8 ± 5.2 dB, n = 6. A horizontal shift in the growth functions is also noted for f2 = 27 kHz. For f2 = 27 kHz, the thresholds are: WT = 40.7 ± 3.7 dB; heterozygotes = 48.3 ± 5.6 dB; ∆IDR KIs = 86.4 ± 2.2 dB SPL. Panel c (**d**) shows average iso-input functions for L1 = 50, L2 = 35 dB (L1 = L2 = 70 dB). The data for WT controls are plotted in red, those for heterozygotes in blue and for KIs in black. Error bars represent standard deviations.

Insertion of a neo cassette into an intron as a component of the targeting vector can influence gene expression^25^. In fact, thresholds were variably elevated in the C1 prestin KI mouse^19^ for both C1 heterozygous and homozygous mice with neo but not in mice when the cassette was removed. Because our initial screening showed that the **∆**IDR heterozygous mice had hearing loss, we removed the neo cassette using the breeding strategy described in the Methods. In the absence of the neo cassette, the homozygous mice continued to show a prestin KO phenotype when screened using DPOAEs at 2f1-f2 and ABRs. Data in Fig. 3 provide DPOAEs for all three genotypes in animals 3–5 weeks of age. The panels at the top show growth or input-output functions for f2 = 12 kHz (Fig. 3a) and f2 = 27 kHz (Fig. 3b). At the lower f2 frequency, the responses from WT (red) and heterozygous (blue) mice are similar, while the DPOAEs in the homozygotes (black) are shifted horizontally along the abscissa about 40 dB SPL. At the higher f2 frequency, the responses in heterozygotes become more variable and decrease relative to controls. In fact, the DPOAE thresholds at f2 = 27 kHz are statistically higher (Student’s t-test, p = 0.026) than WT as originally shown for prestin heterozygous mice^1^. Specifically, the average DPOAE threshold at f2 = 27 kHz for heterozygous mice was 48.3 ± 5.6 dB compared with 40.7 ± 3.7 dB in WT controls. Iso-input functions are provided at the bottom for L1 = 50 and L2 = 35 dB in panel c and L1 = L2 = 70 dB in panel d. At the lower level, there are no measurable DPOAEs at 2f1-f2 for the homozygous mice and the heterozygotes show smaller magnitudes than WT, especially for high f2 frequencies. At 70 dB, small responses can be recorded from young homozygotes at low f2 frequencies (Fig. 3d). Even at this higher level, the heterozygotes have reduced DPOAEs at high f2 frequencies when compared with controls. ABR thresholds were also obtained. At 12 kHz, the ABR thresholds were: WT = 22.5 ± 2.9 dB n = 4; heterozygotes = 25.1 ± 4.1 dB n = 7; ∆IDR KIs = 69 ± 3.6 dB n = 6. At 27 kHz, the ABR thresholds were: WT = 21 ± 2.3 dB; heterozygotes = 25.7 ± 8.2 dB; ∆IDR KIs = 72.3 ± 4.1 dB. When compared to the WT controls, the homozygotes had a threshold shift of 46.5 dB at 12 kHz and a shift of 51.3 dB at 27 kHz. Although heterozygotes had higher ABR thresholds than controls they were not statistically significant (Student’s t-test p = 0.269 at 12 kHz and p = 0.233 at 27 kHz). These results were obtained from WT, heterozygous and homozygous mice obtained from early-generation, heterozygous matings.

### Basal loss of OHCs in the ∆IDR KI mouse

**Figure 4:**
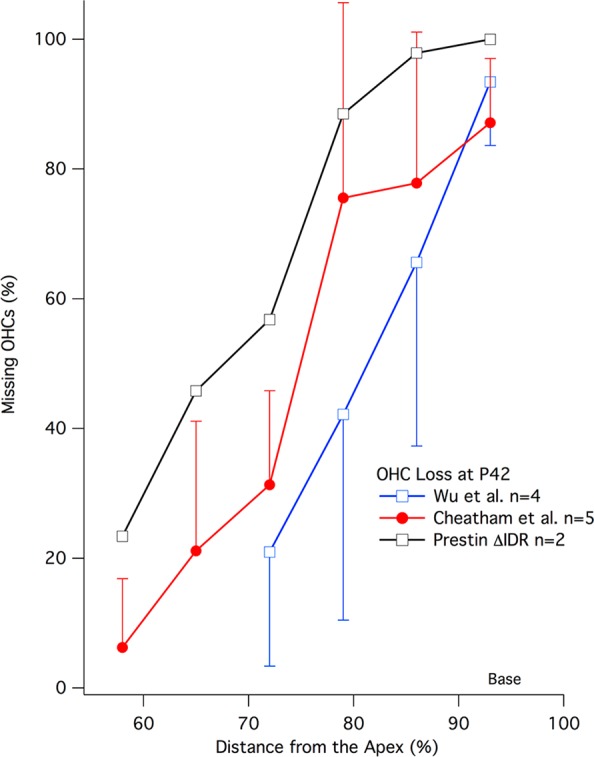
Loss of OHCs in the ∆IDR KI mouse. Cytograms showing the average percent missing OHCs plotted as a function of distance from the apex. The original Wu *et al*.^26^ data are in blue, our confirmation of those results is in red and the ∆IDR prestin results are in black. All mice show loss of OHCs at the base of the cochlea at P42. Error bars represent standard deviations.

Because *in vivo* measurements showed a prestin knockout (KO) phenotype, we examined cochlear whole mounts to assay OHC survival. As in mice lacking prestin, OHC loss was observed in the basal portion of the cochlea. In contrast, WT animals have only sporadic OHC loss that amounts to less than 2% per cochlea^26^. They are, therefore, not included in Fig. 4, which shows the original Wu *et al*.^26^ data (blue) for mice lacking prestin, as well as our confirmation of those results (red). We also append OHC counts obtained from the **∆**IDR prestin KI mice (black). All of the mice used for the hair cell counts were 42 days of age (P42). The data indicate that the KO-like phenotype in cochlear responses (Fig. 3) is also seen in the anatomy where large numbers of OHCs are missing in the basal region of the cochlea^1^. In other words, prestin KO and ∆IDR prestin KI mice show a similar phenotype for both the anatomy and the physiology.

### Absence of prestin protein in OHCs of the ∆IDR KI mouse

**Figure 5:**
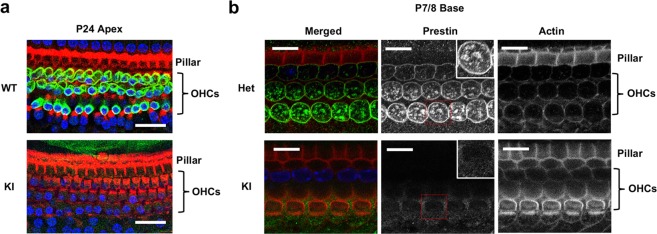
OHCs in prestin ∆IDR KI mice do not express prestin protein. Panel a shows apical cochlear whole mounts from a P24 prestin WT and a ∆IDR KI stained with anti-prestin-N-terminal antisera (green). No prestin signal was observed in KIs (bottom) although OHCs were present as indicated by using phalloidin (red, for actin) and Hoechst (blue, for nuclei) dyes. Scale bars, 25 µm. Panel b shows basal cochlear whole mounts in P7/8 ∆IDR prestin heterozygous and homozygous mice stained as in panel a. During postnatal development, prestin is expressed in cytoplasmic vesicles and at the plasma membrane as seen in the heterozygote. In contrast, prestin protein is completely absent in the ∆IDR KI (“Prestin” panel, insets). At this young age, OHCs are present in apical regions of KI mice as shown in the “Actin” column. Scale bars, 10 µm.

Because the physiological and anatomical results were similar to those in mice lacking prestin, we used antisera containing a prestin antibody that recognizes prestin’s N-terminal to examine protein expression. At P24 in the apex where OHCs are retained, prestin protein is shown by the green staining pattern in WT controls but no staining was observed in ∆IDR prestin homozygous mice (Fig. 5a). Because it is possible that ∆IDR prestin proteins were made but became stalled in the endoplasmic reticulum or Golgi, we also examined prestin expression from P5 to P8. At these early ages, normal animals show that prestin protein predominates in the cytoplasm prior to targeting the membrane^24,27^. However, in ∆IDR prestin homozygous mice, no staining was ever observed in the cytoplasm or at the plasma membrane at P7/8 (Fig. 5b). In contrast, WT prestin staining was found in both the cytoplasm and plasma membrane, as expected. Similar patterns were also found at P5/P6 (data not shown). Cochlear samples were also stained with anti-C-mPrestin^2,28^, which targets the last 20 of prestin’s amino acids, with results like those for anti-N-mprestin. In other words, we did not observe any prestin protein staining in the OHCs of ∆IDR mice regardless of age and/or antibody recognition site. These data are consistent with *in vivo* results, which showed loss of sensitivity in ∆IDR homozygotes (Fig. 3a,b).

### Prestin mRNA is present in OHCs of the IDR KI mouse but it skips exons 17 and 18

**Figure 6:**
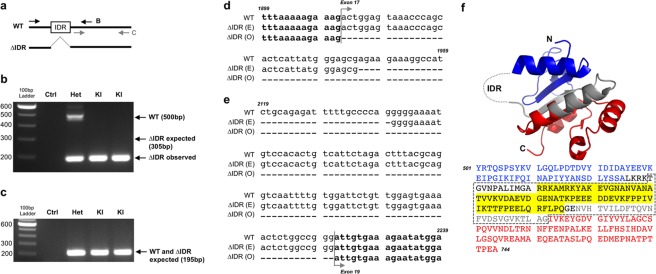
Prestin ∆IDR KI mice express mRNA but it lacks exons 17 and 18. Panel a: PCR primer sets were designed to show mRNA surrounding the IDR, as well as in the region 3′ to the IDR. Panel b: Total RNA isolated from P38 prestin ∆IDR heterozygous and KI mice was used to synthesize cDNA followed by PCR using primer set B that flanks the IDR. Expected product for the prestin WT allele is only observed in the Het lane. Smaller than expected PCR products are observed in both Het and KI lanes for the ∆IDR allele. Ctrl, no cDNA control. Panel c: PCR reactions obtained using the same cDNA from (**b**) but with primer set C that anneals to the region 3′ of the IDR. All bands are of the expected size. Sequence alignments of prestin mRNA at exon 16–17 and 18–19 junctions are shown in panels d and e, respectively. Mouse WT prestin mRNA sequences (NM_030727, WT) from 1899–1959 (**d**) and 2119–2239 (**e**) are shown along with the expected and observed (∆IDR (E) and (O), respectively) ∆IDR sequences. RT-PCR products from ∆IDR KI mice lack all of exons 17 and 18. Panel f: Structure of prestin’s CTD (PDB ID: 3LLO) along with the corresponding mouse prestin sequence (aa 501–744). Residues shown in black are not within the structured regions as they comprise the intervening sequence, which is disordered (aa 556–637, IDR, dotted line). Residues in blue, gray and red correspond to the regions of the structure with the same color. Yellow shading indicates the region targeted for deletion (aa 571–635) in the original design. The boxed region corresponds to residues coded in exons 17 and 18 (aa 560–662).

To understand the lack of prestin protein in ∆IDR mice, we performed RT-PCR to examine prestin mRNA. Figure 6a diagrams two of the several paired primers used to interrogate prestin’s mRNA. Primer pair B surrounds the IDR region targeted for deletion, while primer pair C is restricted to the 3′ region following the deletion. Panel b shows the PCR results obtained for primer pair B. Total RNA was isolated from ∆IDR heterozygous and homozygous mice to synthesize cDNA. The lane for the heterozygous sample has two bands: one for WT at ~500 base pairs (bp) and a second band at ~200 bp. The latter coincides with that in the two homozygous KI samples. This band is smaller than the expected ∆IDR band at 305 bp. Panel c shows results using primer pair C. Both heterozygous and KI samples show a band at 195 bp. This is the expected result as primer pair C targets a region that follows the area targeted for deletion.

We further sequenced the band isolated from KI samples, as shown in Fig. 6b. DNA sequencing data revealed that the deletion in ∆IDR mice was larger than anticipated as both exons 17 and 18 were completely removed. In other words, amino acids 560–662 were deleted, thereby removing virtually all of the IDR region (aa 556–637). Although exon 16 of the ∆IDR allele was expected to splice with the hybrid 17–18 ∆IDR exons, and in turn to join with exon 19 (∆IDR (E) for expected), the PCR products indicate that both exons 17 and 18 (∆IDR (O) for observed) were completely skipped, as shown by the sequence alignment of prestin’s mRNA in Fig. 6d,e. Examination of the boundary between exons 16 and 17 in panel d shows that an additional 33 bp (11 aa) were removed upstream of the target. The mapping in Fig. 6e indicates the junction between exons 18 and 19. Again, the observed PCR product shows a total deletion of exon 18, i.e., the removal of an extra 81 bp (27 aa) downstream of the area targeted for deletion. These sequencing results confirm that the joint exon16-exon19 does not cause a frame shift. The structural model in Fig. 6f shows prestin’s CTD, as well as the mouse prestin aa sequence from 501–744. Residues in the sequence correspond to those in the structure with the same color code. The boxed area indicates the residues coded by exons 17 and 18 that were removed from the ∆IDR prestin mRNA. Notice that the area highlighted in yellow, the targeted deletion, is smaller than the region that was in fact removed. Because all of exons 17 and 18 were deleted, the mRNA for the observed ∆IDR allele is smaller than anticipated (Fig. 6b). In order to further assure the integrity of ∆IDR prestin mRNA, we utilized several primer pairs targeted to aa 493–744 and determined that mRNA was present but that the ∆IDR prestin homozygotes lacked exons 17 and 18, thereby attesting to the stability of this C-terminal region of prestin’s mRNA.

### *In vitro* characterization of ∆Ex17-18 prestin

**Figure 7:**
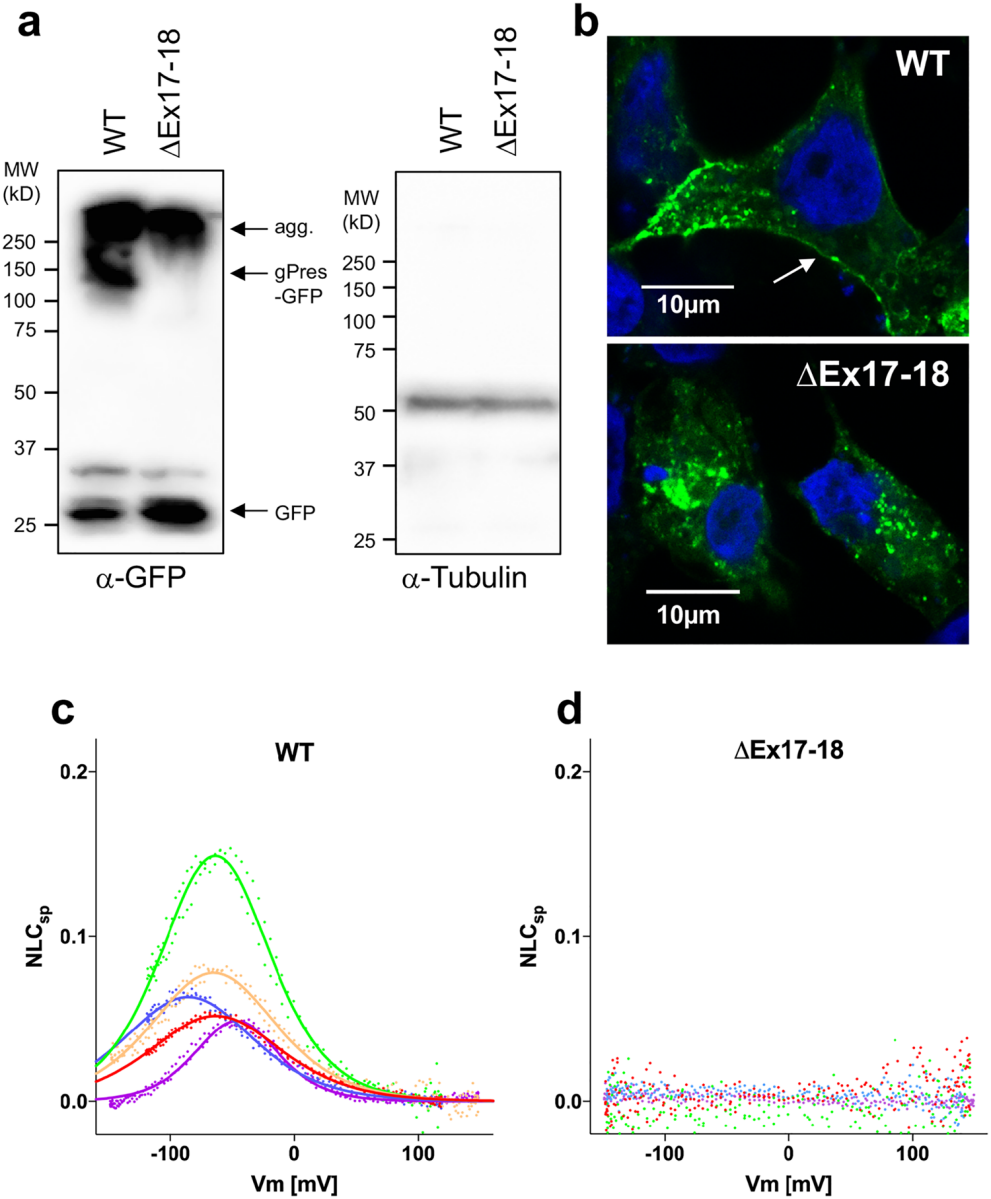
Deletion of exons 17 and 18 results in unstable prestin protein and loss of NLC in HEK293T cells. Panel a: GFP-tagged wild-type prestin (WT) and the prestin mutant that lacks exons 17 and 18 (∆Ex17-18) were transiently expressed in HEK293T cells. Prestin variants were detected using JL8 anti-GFP and tubulin was used as a loading control. (agg: aggregate). Panel b: Representative images of HEK293T cells transiently transfected with EGFP-tagged WT and ∆Ex17-18 prestin. WT prestin localizes to the plasma membrane (white arrow), while ∆Ex17-18 prestin is retained in the cytoplasm. Scale bars, 10 µm. Panel c: HEK293T cells transfected with WT prestin-EGFP show NLC. The data are plotted as specific NLC $({{\rm{NLC}}}_{{\rm{sp}}}\equiv ({{\rm{C}}}_{{\rm{m}}}-{{\rm{C}}}_{{\rm{lin}}})/{{\rm{C}}}_{{\rm{lin}}}$) because larger cells tend to express greater amounts of prestin in their cell membranes. Note that this metric has no dimension. Panel d: In contrast to WT, HEK293T cells transfected with ∆Ex17-18 prestin-EGFP exhibit no sign of NLC. Results in panels C and D were acquired two-days post transfection for both constructs.

The fact that the observed mRNA showed a larger deletion than expected prompted us to create and characterize ∆Ex17-18 prestin so that *in vitro* and *in vivo* results could be more directly compared. In these experiments, HEK293T cells were transfected with WT prestin-GFP or ∆Ex17-18 prestin-GFP-expressing plasmids. Although full-length WT prestin was observed, there was no comparable band for ∆Ex17-18 prestin protein, as shown in the Western blots in Fig. 7a. Both, however, showed aggregation at higher molecular weights, as well as smaller degradation fragments. Protein expression was also confirmed microscopically using the fluorescence of GFP attached to the C-terminus of the WT and ∆Ex17-18 constructs. Figure 7b shows that WT prestin appears at the plasma membrane of HEK293T cells, as shown by the white arrow. In contrast, there was no evidence of membrane targeting for ∆Ex17-18 prestin as fluorescence was restricted to the cytoplasm. The gating charge movement of WT and ∆Ex17-18 prestins was also examined. Although WT prestin exhibits NLC (Fig. 7c), HEK293T cells expressing ∆Ex17-18 prestin show no detectable NLC (Fig. 7d). Because of the large variation in HEK293T cell size, the data are plotted as specific NLC (NLC_sp_, defined in the figure legend) to facilitate comparisons. Taken together, these results suggest that ∆Ex17-18 prestin is unstable. In other words, mutant prestin only appears as aggregated protein within the cytoplasm of HEK293T cells when its constitutive expression is driven by a strong promotor, which in our case is CMV. These observations imply that this unstable protein is most likely quickly degraded in OHCs as no prestin protein was detected (Fig. 5). However, even if ∆Ex17-18 prestin were to be expressed in OHCs, NLC would not be anticipated (Fig. 7).

### The ΔIDR construct is predicted to increase the likelihood of exon skipping resulting in complete loss of prestin exons 17 and 18

The observed lack of the hybrid ΔIDR exon suggests a mis-splicing event *in vivo*. Although several isoforms are reported for prestin^29^ in humans, none of them involve exon 17 and 18 skipping. Thus, naturally occurring alternative splicing is unlikely to be the source of our observation. Since core splicing signals such as the 5′-acceptor site, the 3′-donor site, the polymyrimidine tract (PPT) and the branch site located upstream of the PPT for our hybrid ΔIDR exon are intact, we analyzed the sequence of the hybrid ΔIDR exon for *cis*-regulatory elements such as exonic splicing enhancers (ESE) and silencers (ESS) and compared the results to WT exons 17 and 18. Two online prediction tools, RESCUE-ESE and EX-SKIP, use mouse data bases^30^. RESCUE-ESE^31^ provides information about the likelihood of exon skipping by tracking ESE that promote exon inclusion. By entering the WT exon 17 sequence, we obtained an output of 20 unique ESE matches and 49 unique ESE matches for WT exon 18. In contrast, the hybrid ΔIDR exon resulted in only 9 unique matches. These results were relatively similar to those obtained using EX-SKIP. This tool predicted a total of 119 ESEs for WT exon 17 and 235 for WT exon 18. The hybrid ΔIDR prediction was 76. EX-SKIP^32^ also predicts ESS sites that inhibit exon inclusion, i.e., 28 ESSs for WT exon 17, 68 for WT exon 18 and 57 for the hybrid ΔIDR exon. Hence, the silencer/enhancer ratio, ESS/ESE, was 0.24 for the WT exon 17, 0.29 for the WT exon 18 and 0.75 for the hybrid ΔIDR exon. The higher silencer/enhancer ratio for the hybrid ΔIDR exon is more likely to foster exon skipping when compared to either the WT exon 17 or the WT exon 18. Because our hybrid ΔIDR design also deletes intron 17, containing 842 base-pairs and located between exons 17 and 18, we used ACEScan2^30^ and identified 132 intronic splicing enhancer (ISE) sequences in mice, including 96 unique matches. As ISE can increase the likelihood that a nearby splice site will be used, lack of these elements in our ΔIDR prestin mouse model may have influenced regional splicing, resulting in the exclusion of both exons 17 and 18. Taken together, these results imply that the hybrid ΔIDR exon has a higher likelihood for exon skipping, consistent with our observations.

**Figure 8:**
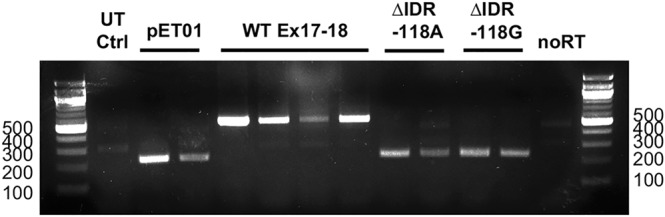
The hybrid ∆IDR exon is not incorporated into mature mRNA. Gel electrophoresis results are shown for the pET01 exon-trap vector alone (lanes 2–3), and for WT (lanes 4–7), ∆IDR with a G > A mutation (∆IDR -118A, lanes 8,9) and corrected ∆IDR constructs (∆IDR -118G, lanes 10–11). Results from untransfected control (UT Ctrl) HEK293T cells appear in lane 1, while the no reverse transcriptase (no RT) control is in lane 12. A 100 bp ladder is appended on both the left- and right-hand sides.

Although various algorithms are available on-line to identify regulatory elements, splicing events are often context dependent and involve a multitude of factors. Therefore, in order to learn how the complete lack of prestin exons 17 and 18 might relate to changes in splicing, mini-genes of WT and the hybrid exon 17–18 (ΔIDR prestin) were created and studied *in vitro* similar to the procedure reported by Booth and colleagues^33^. The depth of intron assessment covered 150 base pairs into the intron separating exons 16 and 17 and 150 base pairs into the intron separating exons 18 and 19. HEK293T cells transfected with the pET01 vector and WT constructs had correctly spliced products with bands at 241 bp and 550 bp, respectively (Fig. 8). In contrast, cells transfected with the ΔIDR construct produced bands that were exactly the same size as the vector alone at 241 bp, indicating a failure to incorporate the hybrid ∆IDR exon. If the correct product had been formed, a band would be expected at 355pb for the ΔIDR prestin construct. Since all ΔIDR prestin mice tested (five homozygotes from 2 litters and one heterozygote), but not the WT control, had nucleotide changes (G to A) at a location 118 base pairs upstream of the beginning of exon 17, we repeated the experiment with this mutation corrected and obtained the same result. In other words, the complete loss of exons 17 and 18 was confirmed even when no variants occurred within the 150 base-pair intronic region in front of exon 17. Untransfected cells produced no bands as did the control sample with no reverse transcriptase (RT). It is possible that additional mutations went undetected as we did not sequence homologous arms in the recombinant ES cell clone. However, given the replication of exon skipping with shorter sequences in the *in-vitro* data, this outcome is unlikely. Therefore, the splicing issue linked to the hybrid ∆IDR exon appears to stem from the partial lack of exons 17 and 18 themselves and/or from within the intron separating exons 17 and 18. Finally, we acknowledge that sequences downstream of the Nneo cassette between the 3′ loxP site and the beginning of the hybrid exon17–18, i.e., part of the intron sequence between exons 16 and 17, could contribute to the exon skipping, a possibility to be considered in future analyses.

## Discussion

Given the ∆IDR prestin mRNA signal, lack of protein in OHCs was perplexing. Although there are several surveillance mechanisms that degrade mRNA to avoid production of toxic proteins^34^, the underlying molecular mechanisms are not understood. Because there is no premature stop codon in ∆IDR mice, we did not expect to see nonsense-mediated mRNA decay, nonsense-mediated translational repression, nonsense-associated alternative splicing, or nonsense-mediated transcriptional gene silencing^35^. In fact, we observed stable prestin mRNA in ∆IDR homozygotes. In addition, non-stop decay is not an option since ΔIDR mRNA retains its usual termination code. Theoretically, translation-dependent surveillance pathways, such as the no-go decay (NGD) pathway^36^, could degrade prestin mRNAs and cause ribosomes to stall during elongation. However, stable ∆IDR prestin mRNA was present in OHCs (Fig. 6). In addition, if this no-go decay pathway were preventing protein from exiting the ribosome, one should see punctate staining in the perinuclear region and this was not found (Fig. 5). Even using both NTD and CTD prestin antibodies, no protein was observed, as early as P5/6. At this age, one usually sees prestin staining within the cell prior to membrane targeting^24,27^. Because the original, intended construct targeted the membrane and inferred NLC in HEK293T cells (Fig. 1), one speculates that the unintentional deletion of 27 amino acids beyond 637 (portions of strand β3 and the α2 helix shown in gray in Fig. 6f) prevented proper folding of ∆Ex17-18 prestin, resulting in its rapid degradation in cochlear OHCs. Hence, these results provide an *in vivo* validation of the *in vitro* results showing that prestin’s CTD is important for protein folding and for membrane targeting^10^. Assuming that ∆IDR prestin protein does not fold correctly, it could be ubiquitylated and degraded by proteasomes as part of the endoplasmic reticulum associated degradation process, which serves to terminate translation^37,38^.

Pancsa and Tompa^39^ recently reviewed potential roles for the IDR, which was removed in our ΔIDR prestin KI mouse. Their report was motivated by the increasing impression that RNA is not simply a courier of DNA information^40^. In fact, RNA structure can influence protein translation in various ways by sequestering ribosome-binding sites, decreasing translational efficiency or even inhibiting translation. Because of these new RNA functions, there is renewed interest in the IDRs of various proteins. Since the IDR does not have a defined 3D structure, numerous parallel or overlapping functions could occur in this region. The latter implies that any feature encoded by a nucleotide sequence that specifies function at the protein level can also do so at RNA/DNA levels.

In the case of prestin, it is possible that loss of the IDR influenced the splicing process. Correct RNA splicing depends on a myriad of regulatory factors that influence the outcome in a context-dependent manner, a notion often referred to as a “splicing code”^41^. Although the replacement of two prestin exons with one that lacked the IDR looked benign, as the hybrid ΔIDR exon contained intact canonical splicing sites, it resulted in production of a protein variant that was immediately degraded, possibly due to the rearrangement of local *cis*-acting splicing elements. In fact, online prediction tools showed that our prestin ∆IDR hybrid construct increased the likelihood of exon skipping by changing the exon splicing silencer/enhancer ratio in favor of exon skipping, which was experimentally confirmed *in vitro* (Fig. 8). Although splicing issues have recently been recognized in connection with the interpretation and diagnosis of mutations associated with human hearing loss^33^, they are also highly relevant when designing constructs for the creation of mouse models to study hearing loss of genetic origin. Future efforts to create new prestin KI mouse models should include careful evaluation of the effects on splicing using both *in silico* splicing predictions and *in vitro* experimental tools when designing constructs for development.

Given the *in vitro* data in HEK293T cells (Fig. 1) showing that the construct used to create the ΔIDR prestin KI mice showed WT-like kinetics, the *in vivo* results were unexpected; hence, our subsequent examination of the ∆Ex17-18 prestin construct. The observation of mutated protein expression in HEK293T cells suggests that generalizing from results obtained in cell lines transfected with a cDNA of choice is sometimes ill advised. In contrast to the *in vivo* condition, HEK293T cells are transfected with cDNA lacking introns and using a powerful promoter to facilitate protein production. One must, therefore, use caution when extrapolating from results obtained *in vitro* in cell lines to results obtained from OHCs with unique cellular features. Although previous mouse models expressing mutant prestins presented a consistent picture with *in vivo* results replicating those in cell lines^5,20^, this was not the case for ∆Ex17-18 prestin, as no protein was observed in OHCs. What the present results do show is that even if ∆Ex17-18 prestin was expressed in OHCs, this protein would not function as a voltage-dependent motor as it appears to be rapidly cleared via proteolysis.

## Methods

All procedures and protocols were approved by the Institutional Animal Care and Use Committees (IACUC) at both Northwestern University and St. Jude Children’s Research Hospital, and by NIDCD. All methods were carried out in accordance with approved guidelines.

### Construct development and transient transfection

The plasmids encoding flipCBS^11^ were kindly provided by Dr. Navaratnam (Yale University School of Medicine). HEK293T cells were then transfected with the prestin-expressing plasmids using Effectene (Qiagen, Valencia CA), as previously described^42^. The cells were used 24–72 hrs post transfection. All methods were carried out in accordance with relevant guidelines and regulations.

### Patch clamp recording and measurement of NLC

Nonlinear capacitance (NLC) was measured under the whole-cell configuration using a sinusoidal (2.5-Hz, 120–150 mV amplitude) voltage stimulus superimposed with two sinusoidal stimuli (390.6 (f_1_) and 781.3 (f_2_) Hz, 10 mV amplitude). The intracellular solution contained the following (in mM): 150 CsCl, 2 MgCl_2_, 10 EGTA, and 10 HEPES, pH 7.3. The extracellular solution contained (in mM): 150 NaCl, 2 MgCl_2_, 2 CoCl_2_, and 10 HEPES, pH 7.3. Osmolarity was adjusted to 310 mmol/kg with glucose and intracellular pressure kept at 0 mmHg during all recordings. An Axopatch 200 A amplifier (Molecular Devices, Sunnyvale, CA) was used for the whole-cell recordings. Voltage-induced current data were collected using jClamp (SciSoft Company, New Haven, CT)^43^. NLC data were analyzed using the following equation:

$${C}_{m}=\frac{\alpha {Q}_{max}\exp [\alpha ({V}_{m}-{V}_{pk})]}{{\{1+\exp [\alpha ({V}_{m}-{V}_{pk})]\}}^{2}}+{C}_{lin}$$

where α is the slope factor of the voltage-dependence of charge transfer, Q_max_ is the maximum charge transfer, V_m_ is the membrane potential, V_pk_ is the voltage at which the maximum charge movement is attained and C_lin_ is the linear capacitance^9,15,44,45^. In order to facilitate comparisons of NLC measurements in HEK293T cells, the magnitude of NLC (C_m_ − C_lin_) is corrected for cell size (C_lin_) because larger cells tend to express greater amounts of prestin in their cell membranes (NLC_sp_ ≡ (C_m_ − C_lin_)/C_lin_)^46^. For stimulus frequency-dependent NLC measurements, f_1_ was set at 195.3 (f_2_ = 390.6), 390.6 (f_2_ = 781.3), 781.3 (f_2_ = 1563), 1563 (f_2_ = 3125), and 3125 (f_2_ = 6250) Hz, i.e., f_2_ = 2xf_1_.

### Targeting vector design and development of the ∆IDR prestin KI mouse

The mouse strain used for the ∆IDR KI ES cells was 129X1/SvJ (Jax#000691), which was called 129/SvJ prior to 2009. After confirming the construct at the restriction enzyme mapping level using sequencing analysis, the transgenic core unit (TCU) at St. Jude Children’s Research Hospital (Memphis TN) karyotyped homologous recombinant ES cells. A total of 10 recombinant ES cell clones were confirmed by PCR (3′ and 5′) and Southern blot analyses. Among 4 independent clones injected with good karyotypes, all gave chimeras and 2 resulted in germline transmission. All knockin mice reported here were derived from a single ES cell clone. Because the neomycin (neo) resistance cassette used to recognize recombinant ES cells can influence gene/protein expression^47^, we removed the cassette using Ella-Cre mice (Jax #003724). Data were acquired from early-generation heterozygous matings, allowing all three genotypes to be produced in a given litter. Although animals were bred on site, genotyping was out sourced to the molecular diagnostics company, Transnetyx, Inc.

### *In vivo* physiological testing

Individual animals of both sexes were screened between 3 and 5 weeks of age and received a pinna reflex test prior to anesthesia using ketamine/xylazine (120 mg/kg and 10 mg/kg, respectively). Distortion product otoacoustic emissions (DPOAE) were recorded in a sound isolation booth using a custom probe placed close to the eardrum such that sound calibrations were performed on individual animals using SysRes^48^. Iso-input functions (f2/f1 = 1.2) were recorded along with input-output functions for f2 at 12 and 27 kHz, where the level of f1 was 10 dB higher than for f2. DPOAE thresholds were defined as the level of f1 that produced a DPOAE at 2f1-f2 of 0 dB SPL. The data were analyzed using EMAV^49^. Auditory brainstem responses (ABR) were also acquired for tone-burst stimuli at 12 and 27 kHz. Threshold was determined by noting the level at which the waveform disappeared into the noise. For these ABR measurements, an average sound calibration was obtained using a real mouse pinna coupler^50^. All results are provided as means and standard deviations. Details are available in other publications^51,52^.

### Anatomical evaluation

Animals were euthanized at 6 weeks of age (postnatal day p42) using Euthasol (200 mg/Kg, IP) in accordance with the IACUC Euthanasia Policy. After cardiac perfusion and post fixation (2.5% glutaraldehyde/0.1% paraformaldehyde), cochleae were decalcified and then dissected using the Eaton-Peabody cochlear dissection protocol^53^. In order to better visualize the hair cells, cochleae were stained with anti-prestin and with a secondary antibody that is conjugated to peroxidase. Exposure to diaminobenzidine (DAB), the substrate oxidized by the enzyme, produces a brown precipitate that makes the hair cells easier to count. Images were captured using MicroSuite^TM^ FIVE Imaging Software (Olympus) and stitched together using Fiji, an image processing package based on ImageJ. The length of each cochlea was determined at the pillar heads and compared to the average cochlear length for that mouse strain^54^ to assure proper stitching. The imaged tissue was then divided into 7% segments with the remaining 2% at the helicotrema, following the original convention^26^. OHCs were counted by row in each division and then summed to obtain the percent missing OHCs in all rows. Data are plotted as means and standard deviations. Additional descriptions can be found in a previous publication^55^.

### Immunofluorescence

Cochlear whole mounts were prepared as above, except 4% paraformaldehyde was used for the cardiac perfusion and post fixation. In order to detect both WT and ∆IDR prestin, N-terminal prestin antisera (anti-N-mprestin)^10^, or C-terminal prestin antibody (anti-C-mprestin)^10^, was used at 1:1000, followed by goat anti-rabbit Alexa 488 secondary antibody at 1:500 (ThermoFisher; AB 2576217). Alexa 546-conjugated phalloidin (ThermoFisher; AB 2632953) and Hoechst 33342 (ThermoFisher) were also used to stain actin and nuclei, respectively. Images were captured on a Nikon A1R confocal microscope with a Plan Apo 20X objective or Plan Apo 60X oil objective (Nikon) controlled by NIS Element software (Nikon). Additional details are provided in a previous publication^2^.

### Prestin RT-PCR

Total RNA from cochlear samples was prepared immediately after the animals were euthanized by directly putting the extracted cochleae into lysis buffer and processing them using the Absolutely RNA Miniprep Kit (Agilent). Prestin mRNAs were detected by first synthesizing the cDNAs from 0.5 µg of total cochlear RNA using M-MLV Reverse Transcriptase (Promega), followed by PCR reactions using Go Taq Flexi DNA polymerase with the following primers: mPres A19 (5′- CTA TGC AAA TAG CGA CTT GTA TAG CAG CG -3′) and mPres B12 (5′- CCA GGA CTG CAT CGT GGA TAC TGT GGA ACA G -3′) flanking the ∆IDR (primer pair B in Fig. 6b); mPres C679S A (5′- GTA TAT TTA GCA GGA TCC AGC CCA CAA GTT GTG AAT GAC -3′) and mPres B10 Alex (5′- TGC CTC GGG GGT GGT GGG TG -3′) for the region 3′ of the IDR (primer pair C in Fig. 6c).

### Western blot analysis

Two days after transfecting HEK293T cells with either EGFP-gPrestin WT or with EGFP-gPrestin completely lacking exons 17 and 18 (ΔEx17-18), lysates were made by incubating the cell pellet in lysis buffer (50 mM Tris-Cl, pH 7.6, 150 mM NaCl, 1% Triton X-100) supplemented with 1 mM PMSF and 1X proteinase inhibitor cocktail (Sigma) followed by centrifugation. The supernatant was then boiled in SDS sample buffer before loading the hand-cast 8% gel and subsequent transfer to nitrocellulose membranes. For the detection of EGFP-prestin variants, Living Colors® A. v. Monoclonal Antibody (JL-8) (Takara Bio USA; AB 10013427) was used. An anti-tubulin antibody (Sigma; AB 477583) was chosen as the loading control. Images were captured using the Kodak Gel Logic 2200 Imaging System controlled by Carestream MI software (Molecular Bioimaging, Bend OR).

### *In vitro* splicing assay

Genomic DNA from WT and ∆IDR prestin mouse tails was amplified for the regions flanking exons 17-18 (+150 bp before and after) and cloned into a pET01 exon-trap vector (MoBiTec, Göttingen, Germany) containing a polylinker or multiple cloning site (MCS) in between two exons with donor and acceptor sequences. The day before transfection, HEK293T cells were seeded on a 6-well plate so that they were 60–70% confluent at the time of transfection. The pET01 constructs were introduced using Effectene (Qiagen) following the manufacturer’s directions. Total RNA was isolated from each well using an Absolutely RNA miniprep kit (Agilent) and cDNA synthesis was carried out with M-MLV-RT (Promega) following the recommended protocols. An pET01-specific primer (cDNA primer 1, 5′- GATCCACGATGCCGC -3′) was used for the reaction. PCR was performed using GoTaq Flexi DNA polymerase with DNA primer pairs 2 (5′- GATCTGCTTCCTGGCCC-3′) and 3 (5′- GGCCACCTCCAGTGCC -3′) from the exon-trap protocol^33^.

### Statistical analyses

The Student’s t-test was used for comparisons between two groups. One-way analysis of variance^56^ combined with the Tukey-Kramer test was used for multiple comparisons. *P* < 0.05 was considered statistically significant. The data are plotted as mean values ± one standard deviation (SD).

## Data Availability

The datasets generated during and/or analyzed during the current study are available from the corresponding author on reasonable request.

## References

[1] Liberman MC (2002). Prestin is required for electromotility of the outer hair cell and for the cochlear amplifier. Nature.

[2] Dallos P (2008). Prestin-based outer hair cell motility is necessary for mammalian cochlear amplification. Neuron.

[3] Cheatham, M. *et al*. Cochlear function in prestin knockout mice. *J Physiol* 560, 821–830 (2004). 10.1113/jphysiol.2004.069559 PMC1665294 15319415

[4] Zheng J (2000). Prestin is the motor protein of cochlear outer hair cells. Nature.

[5] Sharma AK, Rigby AC, Alper SL (2011). STAS domain structure and function. Cell Physiol Biochem.

[6] van der Lee R (2014). Classificiation of intrinsically disordered regions and proteins. Chem Rev.

[7] Ko SB (2004). Gating of CFTR by the STAS domain of SLC26 transporters. Nat Cell Biol.

[8] Fong Peying (2012). CFTR–SLC26 transporter interactions in epithelia. Biophysical Reviews.

[9] Homma K (2010). Interaction between CFTR and prestin (SLC26A5). Biochim Biophys Acta.

[10] Zheng J (2005). The C-terminus of prestin influences nonlinear capacitance and plasma membrane targeting. J Cell Sci.

[11] Bai JP, Navaratnam D, Samaranayake H, Santos-Sacchi J (2006). En block C-terminal charge cluster reversals in prestin (SLC26A5): effects on voltage-dependent electromechanical activity. Neurosci Lett.

[12] Zhang Y, Moeini-Naghani I, Bai JB, Santo-Sacchi J, Navaratnam D (2015). Tyrosine motifs are required for prestin basolateral membrane targeting. Biology open.

[13] Lolli G, Pasqualetto E, Costanzi E, Bonette G, Battistutta R (2016). The STAS domain of mammalian SLC26A5 prestin harbours an anion-binding site. Biochem J.

[14] Bai JP (2010). Prestin surface expression and activity are augmented by interaction with MAP1S, a microtubule-associated protein. J Biol Chem.

[15] Keller JP (2014). Functional regulation of the SLC26-family protein prestin by calcium/calmodulin. J Neurosci.

[16] Guinan JJ (2010). Cochlear efferent innervation and function. Current opinion in otolaryngology & head and neck surgery.

[17] Hackney CM, Mahendrasingam S, Penn A, Fettiplace R (2005). The concentrations of calcium buffering proteins in mammalian cochlear hair cells. J Neurosci.

[18] Tong B (2016). Oncomodulin, an EF-hand Ca2+ buffer, is critical for maintaining cochlea function in mice. J Neurosci.

[19] Gao J (2007). Prestin-based outer hair cell electromotility in knockin mice does not appear to adjust the operating point of a cilia-based amplifier. Proc Natl Acad Sci USA.

[20] Brownell WE, Bader CR, Bertrand D, de Ribaupierre Y (1985). Evoked mechanical responses of isolated cochlear outer hair cells. Science.

[21] Podgorski, M., Philpott, C., Nourse, A., Kriwacki, R. & Zuo, J. Dynamic conformational features of prestin’s C-terminus. *Abs. Assoc. Res. Otolaryngol* (2008).

[22] Seymour ML (2016). Membrane prestin expression correlates with the magnitude of prestin-associated charge movement. Hear Res.

[23] Fang J (2012). Outer hair cell-specific prestin-CreERT2 knockin mouse lines. Genesis.

[24] Yamashita T (2015). Outer hair cell lateral wall structure constrains the mobility of plasma membrane proteins. PlosS Genetics.

[25] Meyers E, Lewandoski M, Martin G (1998). An Fgf8 mutant allelic series generated by Cre- and Fpl-mediated recomibination. Nat Genet.

[26] Wu X, Gao J, Guo Y, Zuo J (2004). Hearing threshold elevation precedes hair-cell loss in prestin knockout mice. Brain Res Mol Brain Res.

[27] Legendre K, Safieddine S, Kussel-Andermann P, Petit C, El-Amraoui A (2008). alphaII-betaV spectrin bridges the plasma membrane and cortical lattice in the lateral wall of the auditory outer hair cells. J Cell Sci.

[28] Cheatham MA (2005). Cochlear function in mice with only one copy of the prestin gene. J Physiol.

[29] Liu XZ (2003). Prestin, a cochlear motor protein, is defective in non-syndromic hearing loss. Human Molecular Genetics.

[30] Yeo G, Hoon S, Venkatesh B, Burge CB (2004). Variation in sequence and organization of splicing regulatory elements in vertebrate genes. Proc Natl Acad Sci USA.

[31] Fairbrother WG, Yeh RF, Sharp PA, Burge CB (2002). Predictive identification of exonic splicing enhancers in human genes. Science.

[32] Raponi M (2011). Prediction of single-nucleotide substitutions that result in exon skipping: identification of a splicing silencer in BRCA1 exon 6. Hum Mutat.

[33] Booth KT (2018). Exonic mutations and exon skipping: Lessons learned from DFNA5. Human Mutation.

[34] Garneau N, Wilusz J, Wilusz C (2007). The highways and byways of mRNA decay. Nat Rev Mol Cell Biol.

[35] Hwang J, Kinm Y (2013). When a ribosome encounters a premature termination codon. BMB Rep.

[36] Doma M, Parker R (2006). Endonucleolytic cleavage of eukaryotic mRNAs with stalls in translation elongation. Nature.

[37] Goldberg AL (2003). Protein degradation and protection against misfolded or damaged proteins. Nature.

[38] Olzmann, J., Kopito, R. & Christianson, J. The mammalian endoplasmic reticulum-associated degradation system. *Cold Spring Harbor Perspectives in Biology* **5** (2013). 10.1101/cshperspect.a013185 PMC3753711 23232094

[39] Pansca R, Tompa P (2016). Coding regions of intrinsic disorder accommodate parallel functions. Trends Biochemical Sciences.

[40] Mauger D, Siegfried N, Weeks K (2013). The genetic code as expressed through relationships between mRNA stucture and protein function. FEBS Lett.

[41] Wang Z, Burge CB (2008). Splicing regulation: from a parts list of regulatory elements to an integrated splicing code. RNA.

[42] Zheng J, Long KB, Shen W, Madison LD, Dallos P (2001). Prestin topology: localization of protein epitopes in relation to the plasma membrane. Neuroreport.

[43] Santos-Sacchi J, Kakehata S, Takahashi S (1998). Effects of membrane potential on the voltage dependence of motility-related charge in outer hair cells of the guinea-pig. J Physiol.

[44] Homma K, Dallos P (2011). Dissecting the electromechanical coupling mechanism of the motor-protein prestin. Communicative & integrative biology.

[45] Homma K, Duan C, Zheng J, Cheatham MA, Dallos P (2013). The V499G/Y501H mutation impairs fast motor kinetics of prestin and has significance for defining functional independence of individual prestin subunits. J Biol Chem.

[46] Kuwabara MF (2018). The extracellular loop of pendrin and prestin modulates their voltage-sensing property. J Biol Chem.

[47] Yamashita T (2012). Normal hearing sensitivity at low-to-middle frequencies with 34% prestin-charge density. PloS one.

[48] Neely, S. & Stevenson, R. SysRes. Technical Memo No. 1, Boys Town National Research Hospital, Omaha NE (1992).

[49] Neely, S. & Liu, Z. EMAV: Otoacoustic emission averager. Technical Memo No. 17, Boy’s Town National Research Hospital, Omaha NE (1994).

[50] Pearce M, Richter CP, Cheatham MA (2001). A reconsideration of sound calibration in the mouse. J. Neurosci. Methods.

[51] Cheatham MA (2014). Loss of the tectorial membrane protein CEACAM16 enhances spontaneous, stimulus-frequency, and transiently evoked otoacoustic emissions. J Neurosci.

[52] Cheatham MA (2016). Increased Spontaneous Otoacoustic Emissions in Mice with a Detached Tectorial Membrane. J Assoc Res Otolaryngol.

[53] Liberman LD, Suzuki J, Liberman MC (2015). Dynamics of cochlear synaptopathy after acoustic overexposure. J Assoc Res Otolaryngol.

[54] Keiler S, Richter CP (2001). Cochlear dimensions obtained in hemicochleae of four different strains of mice: CBA/CaJ, 129/CD1, 129/SvEv and C57BL/6J. Hear Res.

[55] Cheatham M (2015). Prestin-dependence of outer hair cell survival and partial rescus of our hair cell loss in Prestin^V499G/Y501H^ knockin mice. PloS one.

[56] Maison SF, Casanova E, Holstein GR, Bettler B, Liberman MC (2009). Loss of GABAB receptors in cochlear neurons: threshold elevation suggests modulation of outer hair cell function by type II afferent fibers. J Assoc Res Otolaryngol.

